# The effectiveness of email-based exercises in promoting psychological wellbeing and healthy lifestyle: a two-year follow-up study

**DOI:** 10.1186/s40359-016-0125-4

**Published:** 2016-05-17

**Authors:** Minna Torniainen-Holm, Maiju Pankakoski, Tuomas Lehto, Osmo Saarelma, Pekka Mustonen, Kaisla Joutsenniemi, Jaana Suvisaari

**Affiliations:** Mental Health Unit, National Institute for Health and Welfare, Helsinki, Finland; Institute for Molecular Medicine Finland FIMM, University of Helsinki, P.O. Box 30, FIN-00271 Helsinki, Finland; Duodecim Medical Publications Ltd, Helsinki, Finland; Department of Psychiatry, The Hospital District of Helsinki and Uusimaa, Peijas Hospital, Vantaa, Finland

**Keywords:** Web-based, Online, Intervention, Happiness, Wellbeing

## Abstract

**Background:**

Web-based interventions provide a possibility to enhance well-being in large groups of people. Only a few studies have studied the effectiveness of the interventions and there is no information on the sustainability of the effects. Study aims were to investigate both the short (2-month) and long-term (2-year) effects of email-based training for mental health and lifestyle.

**Methods:**

Persons who completed an ‘Electronic Health Check’, as advertised in a TV program, were offered a chance to participate in email-based interventions. The baseline questionnaire was completed by 73 054 people, with 42 761 starting interventions, and 16 499 people participating in at least one of the follow-ups. Persons who did not choose to start the interventions served as controls.

**Results:**

At baseline, the intervention group had a higher level of stress and lower gratitude and confidence in the future than the control group. Both groups showed improvement in the level of stress, but improvement was more marked in the intervention group (*P* < .001 for both time points). In confidence in the future and gratitude, people who chose interpersonal interventions showed significant improvements at both time points (*P* < .001), whereas those choosing lifestyle interventions showed improvement only at the 2-month follow-up. Participants who had done the exercises according to instructions had the most sustained improvements in measures of psychological health at the 2-year follow-up. As for lifestyle, people who had started lifestyle interventions increased their exercise (*P* < .001 at both time points).

**Conclusions:**

Internet-based interventions are feasible for mental health promotion and should be available for people interested in improving their psychological well-being and lifestyle.

**Electronic supplementary material:**

The online version of this article (doi:10.1186/s40359-016-0125-4) contains supplementary material, which is available to authorized users.

## Background

Promoting psychological well-being, besides being important in its own right, may also improve psychological resilience, decrease the risk of mental disorders, increase productivity at work and even promote physical health [1–3]. Well-being is not just absence of mental disorders, it is rather a broad concept that includes happiness as well as other factors that make up a good life, and it includes both affective and cognitive processes [4–6]. Lyubomirsky [7] stated that approximately 50 % of happiness is genetically determined and 10 % is determined by circumstances, whereas 40 % of happiness is composed of what a person does or thinks; therefore, this 40 % can be influenced, and in this article we focus on factors that can be influenced.

Research on how to improve well-being has been done especially within the positive psychology tradition [8]. Many simple exercises have been effective in enhancing well-being and increasing resilience to everyday stress, such as exercises to increase gratitude [9], to increase optimism [10, 11], to promote forgiveness [12], to do good deeds [8] and to decrease rumination [13]. Gratitude has been shown to strongly influence subjective well-being, and exercises to increase gratitude have appeared effective [9]. Optimism refers to expecting positive outcomes in the future [10] and is related to happiness, better subjective well-being and better coping in stressful situations [10, 11]. Earlier studies have shown that especially stress and optimism have a strong impact also on physical health [14–17]. Previously our research group has shown strong links between confidence in the future, as part of optimism, and healthy lifestyle [16]. Lyubomirsky et al. have also suggested that interventions to increase well-being may be most successful when participants are self-selected, when they know about the intervention goals, are motivated and make efforts to reach them [18].

The availability of therapists and the scarcity of financial resources, for example, limit the use of traditional face-to-face psychotherapeutic interventions for improving psychological well-being. The internet provides a venue for improving psychological well-being in larger populations with easy access and low requirements for financial and personnel resources. Several randomized controlled trials and meta-analyses have provided support for the use of internet-based therapy, with or without therapist contact, in the treatment of various psychiatric conditions, including depression, anxiety disorders or occupational stress [19–21]. Internet-based interventions on stress reduction comprise a wide variety of exercises, from mindfulness-based exercises to time management, and these interventions have mostly appeared effective [22–24]. Several studies have also shown that internet-based healthy lifestyle interventions may be effective [25] in weight management [26], in increasing physical activity [27], in reducing alcohol use [28] and in smoking cessation [29].

A few earlier studies have also provided preliminary support for using web-based solutions in the promotion of well-being in the general population. Seligman et al. [8] showed that three out of five happiness exercises, namely identifying three good things in a day, writing and delivering a letter of gratitude and using signature strengths in a new way, were able to increase well-being, and for two of them the effects were still evident in a 6-month follow-up. In a randomized controlled trial using a cognitive-behavioral tool, Powell et al. [30] showed improvement in well-being in a 12-week follow-up in the intervention group compared to controls. In a study with 435 self-selected adults, both writing about best possible selves and making gratitude lists improved subjective well-being compared to writing to-do-lists, and the effect was maintained in the one-month follow-up [31]. A study with a 6-month follow-up noticed that self-compassion and optimism exercises were able to increase happiness in persons vulnerable to depression [32]. While the studies have provided support for web-based interventions, sample sizes have been relatively small [33, 34]. In addition, because follow-up times have mostly been short, at usually a couple of months up to half an year [8, 30], more information is needed on the sustainability of the intervention effects. Since the effect sizes have not been large in previous studies, the intervention can be meaningful for an individual or on a population level only if the interventions have long-term effects on well-being.

We have previously reported the results of a randomized trial on email-based exercises in happiness, physical activity and readings based on the Finnish Happiness-Flourishing Study (FHFS) [35]. In that study, with approximately 3000 participants at the baseline but with a 60 % attrition rate, there was improvement in psychological well-being and a decrease in depressive symptoms in the happiness exercises group and in the physical activity group, but similar improvement was evident also in the active control group receiving only readings [35]. The authors concluded that email-based exercises appear as a promising new tool for reducing well-being disparities [35].

Since then, a new TV program was started which focused on promoting resilience to daily stressors, optimism and gratitude. In the TV program, five Finnish celebrities received each a coach with expertise in improving well-being, and each of the celebrities had their own episode, which showed their training. The program advertised a website where people were able to fill in a questionnaire on their health, lifestyle and psychological well-being, resulting in a feedback report. The report included an estimate of the average life expectancy and the risk of developing coronary heart disease, stroke or diabetes within next 10 years, as well as a description of one’s life habits that impact on health and ways to influence them. People were then offered a chance to start an email-based intervention intended to enhance well-being and additional exercise programs based on their own preferences.

The general aim of the study was to explore the feasibility of this new, freely accessible intervention for improving wellbeing in the general population. More specifically we investigated the level of interest for this kind of intervention and the effectiveness of the intervention both in the short-term and in the long-term in persons who have by themselves sought the intervention. In addition, we investigated whether adherence to the intervention influenced the effectiveness of and satisfaction with the intervention.

The hypotheses were:

1. Intervention improves wellbeing (operationalized as the level of stress, confidence in the future, and gratitude) both in the short-term and in the long-term when compared to their level before the intervention and to people who filled in the questionnaire without participating in the exercises.
2. The intervention improves health-related habits both in the short-term and in the long-term term when compared to their level before the intervention and to people who filled in the questionnaire without participating in the exercises.
3. Adherence to the exercises improves the effectiveness of the intervention.

## Methods

### Recruitment and study procedure

Participants for the present study were recruited through a reality TV program, where five Finnish celebrities received training from five mental health professionals to promote resilience to daily hassles and adversities, optimism and gratitude, presented from October 2012 to January 2013. Part of the TV program was a freely accessible website, where people could test their health, lifestyle, psychological wellbeing and stress coping. The site was also advertised through the web pages of the Finnish Broadcasting Company, through the public health portal of the Finnish Medical Society/Duodecim Medical Publications Ltd and also through various social media channels (Facebook etc.).

The questionnaire at the website provided a health check report that was sent to the participant’s email if they gave the address. On the website, participants were offered the possibility to participate in the training. The participants were informed that the responses in the questionnaire are used in a study into the effectiveness of the intervention, and the persons who give an email address would be contacted again. When participants were contacted again, they were asked to fill in the questionnaire to produce additional information for the research into the email-delivered training. The participants did not receive any compensation for their participation.

Adults (age 18 years or over) who completed the questionnaire between 10 September 2012 and 2 December 2012 were included in this study sample. All persons who participated in the baseline assessment and who had given permission to be contacted again were emailed and requested to complete a similar online questionnaire two months after the baseline assessment and between 25 August and 18 September 2014. Thus, the final follow-up time was approximately two years from the baseline. The non-responders were reminded of the follow-up surveys once.

The study protocol was approved by the Ethics Committee of the Hospital District of Helsinki and Uusimaa.

### Participants

**Fig. 1:**
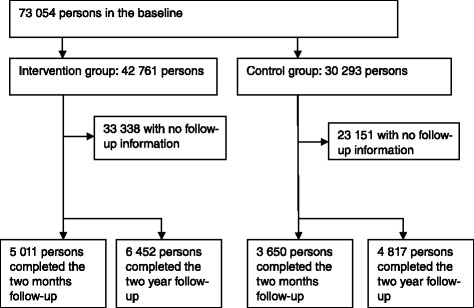
Flow-chart of participation

Altogether 73 054 persons completed the questionnaire of whom 42 761 persons (58.4 %) entered the training (Fig. 1). Persons who did not choose to start the interventions served as controls. The attrition rate in the 2-month follow-up was 88.3 % in the intervention group and 88.0 % in the control group. In the final follow-up, the attrition rate was 84.9 % in the intervention group and 84.1 % in the control group.

### Intervention

The program (Electronic Health and Wellbeing Check and Coaching) is fully owned by Duodecim Medical Publications Ltd./Finnish Medical Society Duodecim, Finland.

Development of the program was funded by the Finnish Funding Agency for Innovation (TEKES). The program was developed from 2010 to 2011 and is continuously updated. The effectiveness of the program has not been previously studied. The program is commercially available in Finland and available through the websites of several health centers and communities.

All participants received an intervention to increase wellbeing and enhance coping with stress, which was based on solution-focused therapy, cognitive behavioral therapy and positive psychology. The intervention included assignments aimed at increasing optimism, decreasing rumination, promoting forgiveness and letting go of past experiences, changing the view on negative experiences and recognizing one’s own coping strategies [7, 8, 14, 36–38].

The participants were sent 17 emails (see Additional file 1: Table S1 for the themes of the emails). Emails included a short paragraph giving some background to the theme, a link to a video motivating to undertake the assignment of the email and instructions for the assignment. Two to three emails were sent in a week and the last email was sent 8 weeks after the first one.

The participants were additionally allowed to choose 1–2 other email-based interventions described in Table 1, which were based on cognitive-behavioral therapy, positive psychology, and health education. The participants received weekly emails relating to these interventions during the same period as they were receiving the wellbeing intervention.

**Table 1:** Description of optional interpersonal and lifestyle interventions used in the study

Content	Key points
Social interactions	Three coaching programs: (i) Positive interaction in a relationship, (ii) Resolving conflicts in a relationship, or (iii) Coaching exercises for families with children. The weekly coaching email message included information, practical advice, and an exercise respectively on each subject based on a cognitive behavioral approach and positive thinking.
Weight management	Weekly email messages consisted of information, practical advice and exercises on weight management (e.g. managing appetite, eating, portion size, and buying food).
Healthy diet	Weekly email messages about healthy diet and practical advice for improvement, and also links to further readings.
Exercise	Weekly email messages aimed at reaching the minimum goal for health promoting physical exercise (at least 2.5 h of brisk physical exercise weekly or 9000 steps daily). Messages included information about health-related physical activity, and practical advice and assignments.
Sleep improvement	Weekly email message containing information, practical advice and exercises on good sleep (e.g. sleep hygiene, environment, and relaxation) and links to further readings.
Alcohol use management	Cognitive behavioral program of two weekly messages to analyze reasons and situations of alcohol use and advice to avoid excessive alcohol use and how to cope with temptations.
Smoking cessation	Cognitive behavioral program of two weekly messages to analyze reasons and situations of smoking, mental exercise, and support for quitting.

### Outcomes

The questionnaire at the freely accessible website included questions related to mental health and lifestyle. As outcome measures, we used the level of stress, two measures of positive mental health, and four lifestyle-related measures that were selected based on our previous research [16]. The questions have been previously used in Finnish population surveys as well as in previous internet-based studies of the research group [16].

Feelings of *stress* were assessed with the item “Have you felt yourself tense, stressed or under strong pressure during the last month?” The question was answered with a 4-point Likert scale where the answer options were (1) “not at all”, (2) “yes, to some extent, but not more than people in general”, (3) “yes, considerably more than people in general”, (4) “yes, my life situation is almost unbearable”.

*Confidence in the future* was assessed with the item “I am very confident about the future.” *Gratitude* was assessed with item “I am very grateful for everything I have received and achieved.” Confidence in the future and gratitude were assessed with a 7-point Likert scale from “definitely agree” to “definitely disagree”.

As secondary outcomes we assessed the following lifestyle-related variables:

(i) Smoking was assessed with the item “Do you smoke currently?” The response categories were “I have never smoked”, “I smoked previously, but I have quit”, “occasionally” and “daily”. The responses were categorized as current daily smoking (yes/no).
(ii) Binge drinking was assessed with the item “How often do you drink alcoholic beverages so that you feel yourself intoxicated?”. The response categories were “less often than once a month”, “at least once a month”, “at least once a week” and “at least a couple of times a week”. The responses were categorized as drinking to intoxication at least once a week (yes/no).
(iii) Exercise was assessed with the item “How much on average do you exercise and do physically demanding activities?”. The response categories were “I usually read, watch television and do activities where I do not move much and that are not physically demanding”, “I walk, bike or otherwise move altogether less than 3 h per week”, “I walk, bike or otherwise move at least 3 h per week”, “I do fitness training like running, jogging, skiing, gymnastics, swimming, ball games, or do physically demanding garden work or something similar on average at least 3 h per week” and “I train for competition regularly many times per week running, orienteering, skiing, swimming, ball games or other physically demanding sports”. The item was categorized as at least three hours of exercise per week (yes/no).
(iv) Diet was assessed with two items. The first was “How much on average do you eat fresh vegetables (one portion is about 70–80 g)?”, and the response categories were “less often than once a day”, “1–2 portions per day”, “3–4 portions per day” and “5 or more portions every day”. The other item was “How much on average do you eat fresh fruits or berries (one portion, for example one apple, is about 130 g)?”, and the response categories were “less often than once a week”, “every week but not every day”, “1 portion per day” and “2 portions or more every day”. We used daily consumption of vegetables or fruits (yes/no) as an indicator of healthy diet.

### Engagement and satisfaction with the intervention

In the 2-year follow-up, we asked whether the participant had done the exercises as instructed, whether the exercises had been easy to understand and whether the intervention had been helpful. These questions were answered on a 7-point Likert scale from “definitely agree” to “definitely disagree”. In addition, the respondents were asked whether they would recommend the intervention to other people (yes/no/don’t know).

### Statistical analyses

Baseline comparisons between respondents who chose the intervention and those who only filled in the questionnaire were done with the t-test for continuous or ordinal variables and with the *χ*^2^-test for categorical variables. Effect sizes were calculated with Eta-squared for continuous or ordinal variables and with Cramer’s Phi for categorical variables. Drop out at the 2-month and 2-year follow-ups was analyzed using logistic regression. Bayesian model averaging was used to determine the predictors of missing values [39].

Almost all respondents who chose the intervention had also chosen additional interventions. Therefore, we analyzed the effects of lifestyle interventions (Alcohol use management, Smoking cessation, Weight management, Exercise, Healthy diet, Sleep) and interpersonal interventions (Coaching exercises for families with children, Positive interaction in relationship, Resolving conflict in relationship) separately. Respondents who had not chosen any additional interventions (66 persons with follow-up data) were excluded from the analysis because their small number did not permit reliable estimation of the effect of the wellbeing-targeted intervention only. Intervention effects were analyzed only for those subjects who had completed at least one of the follow-up questionnaires (*N* = 16 499).

Changes in the outcome variables were analyzed using generalized estimating equation (GEE) models that take into account the longitudinal structure of the data [40]. Linear modeling was used for continuous outcomes (confidence in the future, gratitude and stress) and logistic modeling for binary outcomes (binge drinking, smoking, physical exercise and vegetable consumption). The models contained the main effects of time as a categorical variable and intervention type (lifestyle and interpersonal) and intervention-time interactions. Age, gender and education years were controlled for. The effect of adherence to the exercises within the intervention group was also analyzed using GEE modeling.

All analyses were performed using the R-program version 3.1.1. [41].

## Results

### Characteristics of the sample

Altogether, 42 761 persons started interventions, and 16 499 persons participated in at least one of the follow-ups. At the baseline, participants choosing the intervention (hereafter the intervention group) were slightly younger, had more years of education, were more often employed and in a relationship, and had less confidence in the future, less feelings of gratitude and more stress than those who only filled in the questionnaire (hereafter called the control group). The intervention group had less binge drinking and daily smoking, and they consumed vegetables and/or fruits daily more often than the control group, but they were physically less active. Women chose the intervention more often than men, which may explain part of these differences. The effect sizes of the differences between the intervention and control groups were small (Table 2).

**Table 2:** Baseline characteristics of the sample comparing participants who chose the email-based exercise program (intervention group) and participants who only filled in the questionnaire (control group)

	Intervention group (*N* = 42 761)	Control group (*N* = 30 293)	*χ*2 or *t*-test	*P*	Phi or eta squared
Age (mean (SD))	47.5 (13.0)	48.5 (14.4)	−9.76	0.000	0.001
Sex					
Men (*N* (%))	10784 (25.2 %)	9790 (32.3 %)	441.6	0.000	0.078
Women (*N* (%))	31977 (74.8 %)	20503 (67.7 %)			
Education years (mean (SD))	15.3 (3.7)	14.7 (3.9)	23.2	0.000	0.007
Current main activity					
Employed (*N* (%))	34946 (82.6 %)	22840 (76.3 %)	432.8	0.000	−0.077
Other^a^ (*N* (%))	7382 (17.4 %)	7106 (23.7 %)			
In relationship					
Yes (*N* (%))	33213 (78.7 %)	23091 (77.2 %)	22.9	0.000	−0.018
No (*N* (%))	9009 (21.3 %)	6833 (22.8 %)			
Confidence in the future (mean (SD))	5.21 (1.51)	5.34 (1.44)	−11.8	0.000	0.002
Gratitude (mean (SD))	5.69 (1.35)	5.76 (1.30)	−7.51	0.000	0.001
Stress (mean (SD))	2.29 (0.70)	2.16 (0.69)	24.8	0.000	0.008
Binge drinking weekly					
Yes (*N* (%))	3612 (15.3 %)	4836 (14.3 %)	10.1	0.001	−0.013
No (*N* (%))	19996 (84.7 %)	28882 (85.7 %)			
Daily smoking (*N*, %)					
Yes (*N* (%))	4905 (11.6 %)	4190 (14.0 %)	89.0	0.000	−0.035
No (*N* (%))	37395 (88.4 %)	25833 (86.0 %)			
Physical exercise at least 3 h/week					
Yes (*N* (%))	26555 (62.6 %)	19788 (65.9 %)	80.2	0.000	−0.033
No (*N* (%))	15857 (37.4 %)	10260 (34.1 %)			
Daily use of vegetables and/or fruits					
Yes (*N* (%))	35587 (83.4 %)	24833 (82.2 %)	18.8	0.000	0.016
No (*N* (%))	7072 (16.6 %)	5379 (17.8 %)			

^a^Group “Other” includes unemployed, students, retired or those managing their own household or taking care of family members

### Drop out

Of the intervention group, 11.6 % participated in the 2-month and 15.0 % in the 2-year follow-up, while the respective figures for the control group were 11.9 and 15.7 %. Variables predicting drop out at the 2-month follow-up were younger age (OR = 0.98, 95 % CI = 0.98–0.99), male gender (OR = 1.51, 95 % CI = 1.42–1.61), less years of education (OR = 0.98, 95 % CI = 0.97–0.99), binge drinking (OR = 1.2, 95 % CI = 1.1–1.3), daily smoking (OR = 1.41, 95 % CI = 1.29–1.55), doing physical exercise less than 3 h/week (OR = 1.14, 95 % CI = 1.07–1.2), and not eating vegetables and/or fruits daily (OR = 1.17, 95 % CI = 1.08–1.27). Subjects in the intervention group were somewhat more likely to drop out compared to subjects in the control group (OR = 1.10, 95 % CI = 1.04–1.16).

Similar variables were associated with drop out also at the 2-year follow-up: younger age (OR = 0.99, 95 % CI = 0.99–0.99), male gender (OR = 1.35, 95 % CI = 1.28–1.42), less years of education (OR = 0.97, 95 % CI = 0.96–0.98), stress (OR = 0.92, 95 % CI = 0.88–0.95), binge drinking (OR = 1.22, 95 % CI = 1.14–1.32), daily smoking (OR = 1.34, 95 % CI = 1.24–1.46), doing physical exercise less than 3 h/week (OR = 1.13, 95 % CI = 1.07–1.19), not eating vegetables and/or fruits daily (OR = 1.16, 95 % CI = 1.08–1.24) and being in the intervention group (OR = 1.12, 95 % CI = 1.07–1.18).

### Effects of interventions on the primary outcome variables

Of the additional interventions, the most popular were weight management, sleep, and positive interaction in relationship interventions (Additional file 1: Table S2). The means and standard deviations of the groups at different time points in the level of stress, in gratitude, and in confidence in the future can be seen in Table 3 and in Additional file 1: Table S3. At baseline, people who chose interpersonal interventions had a lower level of confidence in the future and gratitude and a higher level of stress but healthier lifestyle than those who chose lifestyle interventions. Note that people who had chosen both lifestyle and interpersonal interventions (*n* = 2237) are included in both groups.

**Table 3:** Outcome variables in the intervention groups and other participants at baseline, 2-month and 2-year follow-ups; baseline results are reported for those who answered to at least one of the follow-up questionnaires (*N* = 16 499)

		Confidence in the future mean (SD)	Gratitude mean (SD)	Stress mean (SD)	Binge drinking weekly *N* (%)	Current smoking *N* (%)	Physical exercise 3 h/week *N* (%)	Daily use of vegetables and/or fruits (*N* (%)
Lifestyle intervention	Baseline (*N* = 7851)	5.34 (1.47)	5.8 (1.31)	2.77 (0.71)	736 (11.96)	703 (9.05)	5042 (64.66)	6795 (86.74)
	2-month follow-up (*N* = 4170)	5.63 (1.35)	6.07 (1.15)	2.94 (0.66)	274 (8.9)	278 (6.76)	2777 (67.19)	3739 (89.86)
	2-year follow-up (*N* = 5362)	5.4 (1.42)	5.92 (1.21)	2.95 (0.66)	350 (8.75)	412 (7.76)	3761 (70.47)	4857 (90.8)
Interpersonal intervention	Baseline (*N* = 3743)	5.0 (1.57)	5.55 (1.41)	2.63 (0.71)	231 (7.97)	211 (5.7)	2577 (69.27)	3291 (88.09)
	2-month follow-up (*N* = 2063)	5.43 (1.4)	5.89 (1.22)	2.85 (0.65)	86 (5.67)	101 (4.96)	1406 (68.69)	1852 (90.17)
	2-year follow-up (*N* = 2531)	5.25 (1.47)	5.8 (1.25)	2.85 (0.66)	132 (6.93)	137 (5.47)	1846 (73.25)	2307 (91.29)
Control group	Baseline (*N* = 7142)	5.42 (1.42)	5.83 (1.25)	2.89 (0.69)	675 (12.17)	777 (10.96)	4897 (69.02)	6065 (85.11)
	2-month follow-up (*N* = 3650)	5.52 (1.36)	5.91 (1.2)	2.96 (0.67)	244 (8.93)	341 (9.42)	2435 (67.21)	3080 (84.48)
	2-year follow-up (*N* = 4817)	5.34 (1.42)	5.87 (1.21)	2.99 (0.68)	317 (8.73)	442 (9.28)	3425 (71.61)	4275 (88.97)

**Fig. 2:**
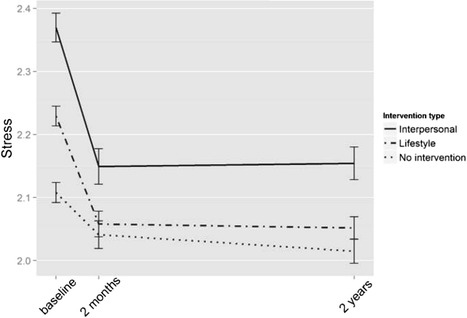
Level of stress in the intervention and control groups over time

The level of stress in the groups at different time points can be seen in Fig. 2 and the means and standard deviations in Table 3 and in Additional file 1: Table S3. Adjusting for the effects of age, sex, and years of education, the time*group interaction was significant both for the group receiving the lifestyle (*P* < 0.001 for both time points) and for the group receiving the interpersonal intervention (*P* < 0.001 for both time points) in the level of stress: the intervention groups had more stress at baseline and also at follow-ups, but they improved more than the control group in the 2-month and 2-year follow-ups. Of note, both intervention groups had received the wellbeing intervention, including assignments to enhance coping with stress.

**Fig. 3:**
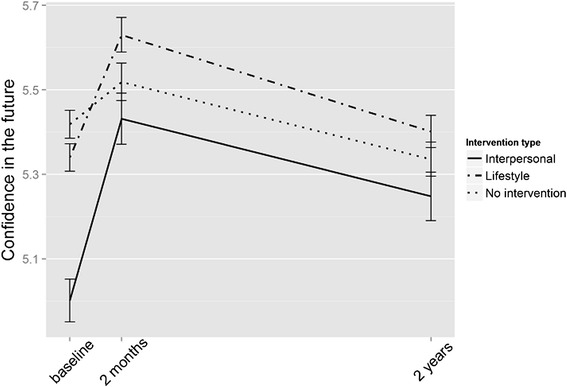
Confidence in the future in the intervention and control groups over time

As shown in Fig. 3, confidence in the future was lower in the intervention groups, especially in the interpersonal intervention group, at baseline than in the control group (see Table 3 and Additional file 1: Table S3 for means and standard deviations). In a similar analysis for confidence in the future, the time*group interaction was significant (*P* < 0.001 for both time points) for the interpersonal intervention group: while their confidence in the future remained lower than in the other two groups, they improved more. In the lifestyle intervention group, the time*group interaction was significant (*P* < 0.001) in the 2-month follow-up, but nonsignificant in the 2-year follow-up (Fig. 3.).

**Fig. 4:**
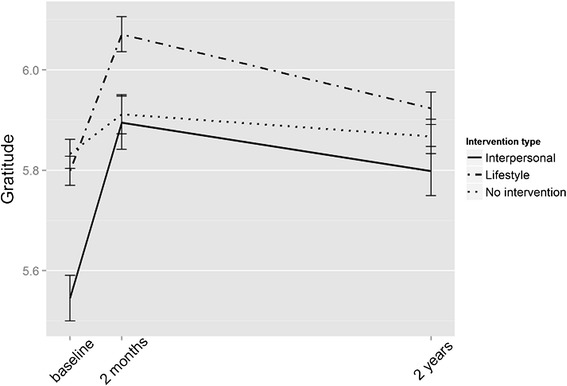
Gratitude in the intervention and control groups over time

Gratitude was lower in the intervention groups in baseline than in the control group (see Table 3 and Additional file 1: Table S3 for means and standard deviations). The time*group interaction was significant (*P* < 0.001 for both time periods) in the interpersonal intervention group, indicating that they improved more than the other groups as can be seen in Fig. 4. The time*group interaction for the lifestyle group was significant at two months (*P* < 0.001) but not at two years (Fig. 4.).

In all three variables, the effect of intervention diminished over time, but the 2-year values for the intervention group were still higher than the baseline values.

### Effects of interventions on lifestyle

When age, sex, and years of education were adjusted for, the only time*group interaction in binge drinking was in the 2-year follow-up for the interpersonal intervention group (*P* = 0.01). The interpersonal intervention group had the lowest level of binge drinking at every time point, but they had increased their frequency of binge drinking between the 2-month and 2-year follow-ups (interpersonal group baseline: 8 %, 2-month follow-up: 6 %, 2-year follow-up 7 %; other groups baseline 12 % and both follow-ups: 9 %; Table 3, Additional file 1: Table S3 and Figure S1.).

Daily smoking decreased somewhat in all groups, with no significant time*group interactions (Table 3, Additional file 1: Table S3 and Figure S2.).

For exercising at least 3 h per week, the time*group interaction was significant for the lifestyle intervention group at both time points (*P* < 0.001), and only they increased their exercise level between both time points (baseline: 65 %, 2-month follow-up: 67 %, 2-year follow-up 70 %). The time*group interaction was also significant for the interpersonal group at two months (*P* = 0.02; baseline and 2-month follow-up: 69 %, 2-year follow-up 73 %), due to a smaller drop in the exercise level in that group than in the control group (baseline: 69 %, 2-month follow-up: 67 %, 2-year follow-up 71 %; Table 3, Additional file 1: Table S3 and Figure S3.)

The time*group interaction was significant for eating vegetables and fruits daily for the lifestyle intervention group at two months (*P* < 0.001). By two years, all groups had improved in eating vegetables and fruits daily (Table 3, Additional file 1: Table S3 and Figure S4).

### Engagement and satisfaction with the intervention

Satisfaction with the intervention and engagement were asked about at the 2-year follow-up. Most participants had not done the exercises as instructed: on a Likert scale scoring of −3 for “definitely disagree” to 3 for “definitely agree”, the average rating of adherence was −0.30 (SD 1.49). For the question assessing whether the exercises had been easy to understand (clarity), the average rating was 0.23 (SD 1.57), and for their helpfulness it was −0.10 (SD 0.39). When the participants were asked whether they would recommend the intervention to others, 43.2 % would and 9.8 % would not recommend the intervention, while 47.0 % were unsure. People who had chosen interpersonal interventions had higher scores in the question assessing clarity (t = −2.89, *P* = 0.004) and helpfulness (t = −2.79, *P* = 0.005) of the exercises.

We analyzed within the intervention group whether adherence to the exercises influenced the outcomes. We combined the Likert scale answers into two groups: adherent (scores 1–3) and non-adherent (scores −3 – 0). Adherence was associated with better 2-year outcome in stress (time*adherence interaction *P* = 0.01; Additional file 1: Figure S5.), confidence in the future (time*adherence interaction *P* < 0.001; Additional file 1: Figure S6.) and gratitude (time*adherence interaction *P* < 0.001; Additional file 1: Figure S7.). Significant interactions were not seen for lifestyle variables.

## Discussion

We used email-delivered training interventions based on solution-focused therapy, positive psychology, cognitive behavioral therapy, and health education, which were offered for people completing an electronic health check. The participants were recruited via a website that was advertised in a reality TV program where celebrities received training to promote resilience to daily hassles and adversities and to increase optimism and gratitude. The control group consisted of people who filled in an electronic health check and received a personalized feedback report at the website, but who did not choose to start any interventions. There was wide interest for both electronic health check and for the interventions. We found that both people starting interventions and the control group showed improvements in psychological health and in lifestyle, but improvement was more marked in the intervention groups. By the 2-year follow-up, these effects were attenuated but still present. Participants who had done exercises according to instructions showed sustained improvement in measures of psychological health in the 2-year follow-up. Our results are comparable to previous studies offering positive psychological interventions via the Internet, many of which have found these interventions effective at least in the short term [8, 18, 30, 33, 34].

The intervention group had a common wellbeing intervention targeting coping with stress, and this seemed to be effective: Both those who had additionally chosen lifestyle-interventions and those who had chosen interpersonal interventions reported lower levels of stress at the 2-month and 2-year follow-ups. The wellbeing intervention also had elements aimed at increasing gratitude and confidence in the future, and both seemed to have had an effect on the intervention group at the 2-month follow-up. The group that in addition had chosen interpersonal interventions had maintained more of that improvement by the 2-year follow-up. This finding accords with the central role of social relationships for positive mental health [42], and also with previous findings of the positive effect of having multiple exercises in an online positive psychological intervention [43].

As for lifestyle, the largest improvements were seen in the lifestyle intervention group in physical exercise and daily use of vegetables or fruits, but all groups had improved by the 2-year follow-up. This accords with previous studies which have suggested that internet-based lifestyle interventions may be effective [25]. Previous studies of internet-based wellness approaches have found more positive results on these variables in non-randomized than in randomized trials [44]. While this may indicate a selection bias, it may also be that motivation has a crucial effect in internet programs targeting lifestyle improvement, as it has in positive psychological interventions [18]. Even assuming that people who had been able to improve their lifestyle were more likely to respond in the follow-up, the sustained improvement in the physical exercise group in the 2-year follow-up was encouraging.

We found that people who reported having done at least part of the exercises according to the instructions had long-lasting improvement in perceived level of stress, gratitude and optimism. This accords with previous research which found that the effortful pursuit of happiness-enhancing web-based interventions improved their effectiveness [18].

Our intervention differs from most previous studies in providing individual emails and combining multimedia platforms to motivated individuals in a wide age-range [45, 46]. With the aim of motivating individuals via perceived autonomy [47], we combined positive psychology interventions with the possibility to choose from other lifestyle interventions, such as tobacco cessation. A number of validation studies have been completed on the individual exercises in our intervention [7, 8, 14, 36–38]. The central premise of the intervention is to address individual positive resources. A combination of exercises is more likely to resonate in individuals than a single exercise that may not appear relevant to some subgroups. Previous studies have successfully combined multiple exercises into effective interventions [34, 48].

The study had limitations. The study groups are not representative of the Finnish adult population. Most of the respondents were women with a relatively high level of education, and their lifestyle was healthier than in the general population on average. For example, less than 15 % were current smokers, compared to 27 % of men and 19 % of women in the general population in 2012 [49]. These characteristics resemble those found by Parks et al. [42] for online happiness seekers. Attrition between the baseline survey and the two follow-ups was large, and it was selective in that people with poorer health habits were less likely to respond in the follow-up. The study was not originally designed as a clinical trial but as a follow-up study. People enrolled in the intervention on the basis of their own interest and were allowed to choose additional interventions freely. It was not possible to study the effects of all possible intervention combinations that the participants had chosen. Therefore, the results of the follow-up should be interpreted with caution. However, it has been suggested that positive psychology interventions may be most successful when participants know about the intervention and commit to it [18], while it has been suggested that using multiple positive activities simultaneously can inhibit adaptation to their hedonic benefits by bolstering variety and novelty [42]. Besides a lack of randomization, another limitation entailed not being able to assess other possible factors that might influence, for example, stress levels and using single items to assess constructs. Moreover, some of the positive effect observed in the control group could be due to the TV-program, another interesting method for influencing positively the mental, physical, and social health of the population.

## Conclusions

To conclude, internet-based interventions are easy to access, there is a potential to reach and engage a large number of people, and the cost related to the programs is smaller than in face-to-face services [50]. In this study, over 70 000 Finns completed the electronic health-check and over 40 000 started interventions, demonstrating that there is interest in these kinds of services. Therefore, the interventions can be cost-effective and are a feasible method of mental health promotion. Mental health promotion should become a public health priority [51] because of the substantial burden related to mental and substance use disorders [52] and because positive psychological wellbeing may also improve physical health [53]. The positive results found in this large observational study suggest that internet-based interventions should be available for people interested in improving their psychological wellbeing and lifestyle.

### Ethics approval and consent to participate

The study protocol was approved by the Ethics Committee of the Hospital District of Helsinki and Uusimaa. The participants were informed that the responses in the questionnaire are used in a study into effectiveness of the intervention, and the persons who give an email address would be re-contacted. When participants were re-contacted, the participants were requested to fill in the questionnaire to produce additional information for the research into the electronical training.

## Availability of data and materials

In research collaboration, data can be shared but sharing requires amendment to the ethics committee permission and a separate agreement with Duodecim Medical Publications Ltd. The ethics committee will evaluate whether the intended collaboration is concordant with the consent given by the participants. Pekka Mustonen (pekka.mustonen@duodecim.fi) at Duodecim Medical Publications Ltd can be contacted.

## Additional file

Additional file 1: Table S1. The themes of the emails.** Table S2.** Participation for different types of interventions. **Table S3.** Outcome variables in the intervention and controls groups at baseline, 2-month and 2-year follow-ups. **Figure S1.** Binge drinking in the intervention and controls groups over time. **Figure S2.** Daily smoking in the intervention and control groups over time. **Figure S3.** The proportion of participants doing physical exercise at least 3 h per week in the intervention and control groups over time. **Figure S4.** The proportion of participants using vegetables or fruits daily in the intervention and control groups over time. **Figure S5.** Level of stress in the intervention group by adherence to the treatment protocol. **Figure S6.** Confidence in the future in the intervention group by adherence to the treatment protocol. **Figure S7.** Gratitude in the intervention group by adherence to the treatment protocol. (PDF 302 kb)
