# Supplementary material for: The effectiveness of email-based exercises in promoting psychological wellbeing and healthy lifestyle: a two-year follow-up study
Source: BMC Psychol. 2016 May 17;4:21. doi: 10.1186/s40359-016-0125-4 (PMC4869346; doi:10.1186/s40359-016-0125-4)

Supplementary Table 1. The themes of the emails.

Email	Theme
1	Observing burdening things in daily life, reflecting on why they are a burden and thinking of solutions.
2	Thinking of a past negative event, reflecting and writing down 1-3 things learned from it, and discussing it with someone.
3	Thinking each evening of three good things in the day, what makes it possible for these things to happen to the person and how these things could happen in future.
4	Thinking of a positive or humorous statement to use when facing adversities that could help in dealing with the situation in a peaceful way, learning to use it and observing the effects.
5	Recognizing coping strategies that helped in a past situation, and how these strategies could help with a current problem.
6	Learning to use automatic thoughts such as ‘luckily nothing worse happened’, which can assist in finding a positive view in difficult situations.
7	Promoting forgiveness by imagining that a person who has hurt the participant has apologized for the event, then deciding to forgive for the participant’s own good and imagining writing a letter to that person.
8	Limiting worrying to a specific 30-minute time in a day.
9	Taking distance from a negative emotion by imagining that the emotion is caused by a creature and giving that creature a name, and thinking, what could get the creature to come close and stay, and what would make that creature go away.
10	The email is started with an example how the consequences of an event that first appear positive or negative may later turn out to be the opposite. Next the participant is encouraged to think with this perspective of a recent negative event.
11	Accepting the unchangeable by imagining meeting a wise person who tells that the thing really cannot be changed, then placing a small object within sight and thinking every time that the participant sees the object, that ‘I just have to live with it’ or ‘I’m not going to let that ruin my life’, and then observing how the method has an effect.
12	Doing 3-5 good deeds for others on one day a week for many weeks.
13	Letting go of a past event that still bothers by writing about it in detail on paper, adding possible missing things the next day and then destroying the paper.
14	Paying attention to negative things happening and imagining that each of them has three positive consequences, and discussing the idea with someone.
15	Writing down 5 things to be grateful for once a week.
16	The participant is asked to imagine meeting a person who goes through a similar difficult situation as the participant, and that the situation has caused strong emotions in the imaginary person. The participant is prompted to think what to say to the persons that would make that person feel better.
17	Summary of the assignments done during training and advice to continue to do exercises.

Supplementary Table 2. Participation for different types of interventions (%) (N=9357)

	All	Lifestyle	Interpersonal
Coaching for families with children	10.6	5.9	26.4
Positive interaction in a relationship	24.9	14.0	62.0
Resolving conflicts in a relationship	13.0	8.8	32.4
Sleep	28.5	34.0	18.8
Healthy nutrition	14.2	17.0	5.6
Physical exercise	22.5	26.8	8.5
Weight management	38.6	46.1	22.7
Controlling alcohol use	13.1	15.6	2.8
Smoking cessation	2.9	3.5	1.4

Supplementary Table 3. Outcome variables in the intervention and controls groups at baseline, 2-month and 2-year follow-ups; baseline results are reported for those who completed at least one of the follow-up questionnaires (N=16499)

	Intervention group			Control group		
	Baseline (N=9357)	2-month follow-up (N=4977)	2-year follow-up (N=6406)	Baseline (N=7142)	2-month follow-up (N=3650)	2-year follow-up (N=4817)
Confidence in the future (mean, SD)	5.28, 1.49	5.61, 1.34	5.38, 1.43	5.42, 1.42	5.52, 1.36	5.34, 1.42
Gratitude (mean, SD)	5.76, 1.32	6.05, 1.15	5.9, 1.22	5.83, 1.25	5.91, 1.2	5.87, 1.21
Stress (mean, SD)	2.25, 0.71	2.07, 0.66	2.07, 0.66	2.11, 0.69	2.04, 0.67	2.01, 0.68
Binge drinking weekly (N, %)	819, 11.25	301, 8.23	397, 8.3	675, 12.17	244, 8.93	317, 8.73
Current smoking (N,%)	757, 8.18	309, 6.29	449, 7.08	777, 10.96	341, 9.42	442, 9.28
Physical exercise (N, %)	6133, 66	3347, 67.85	4565, 71.6	4897, 69.02	2435, 67.21	3425, 71.61
Daily use of vegetables and/or fruits (N, %)	8142, 87.21	4469, 90.03	5818, 91.03	6065, 85.11	3080, 84.48	4275, 88.97

Supplementary Figure 1. Binge drinking in the intervention and controls groups over time

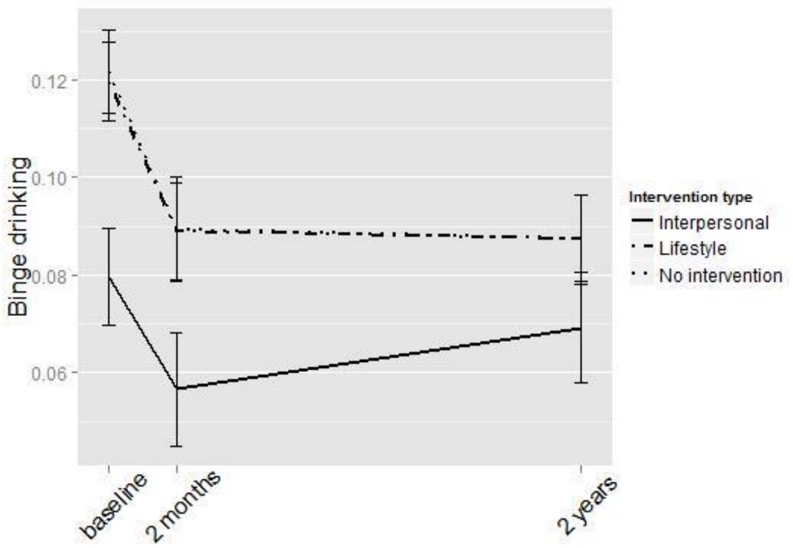

Supplementary Figure 2. Daily smoking in the intervention and control groups over time

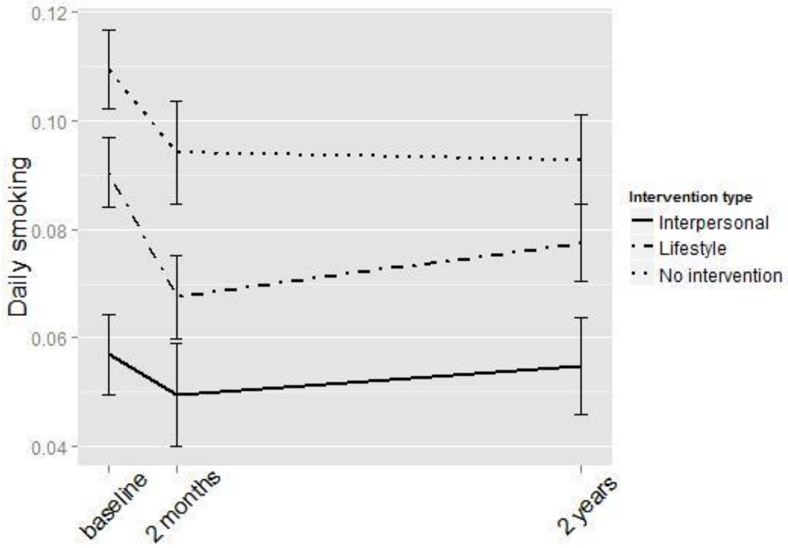

Supplementary Figure 3. The proportion of participants doing physical exercise at least 3 hours per week in the intervention and control groups over time

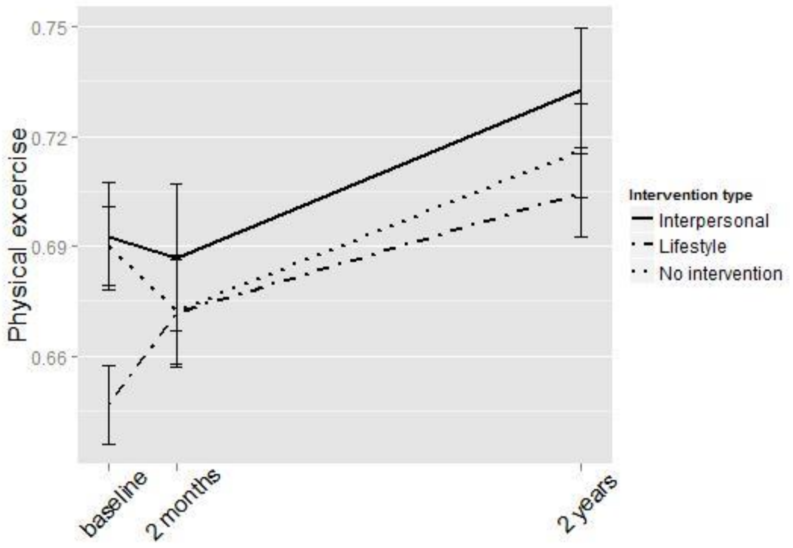

Supplementary Figure 4. The proportion of participants using vegetables or fruits daily in the intervention and control groups over time

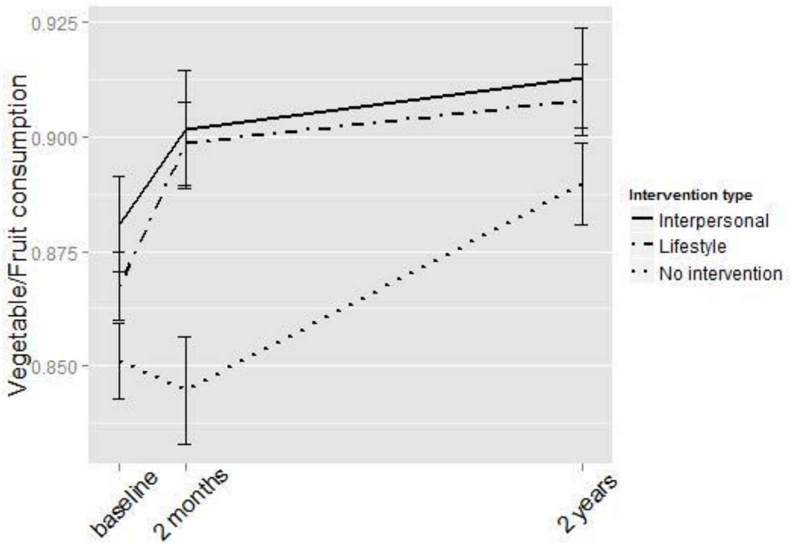

Supplementary Figure 5. Level of stress in the intervention group by adherence to the treatment protocol

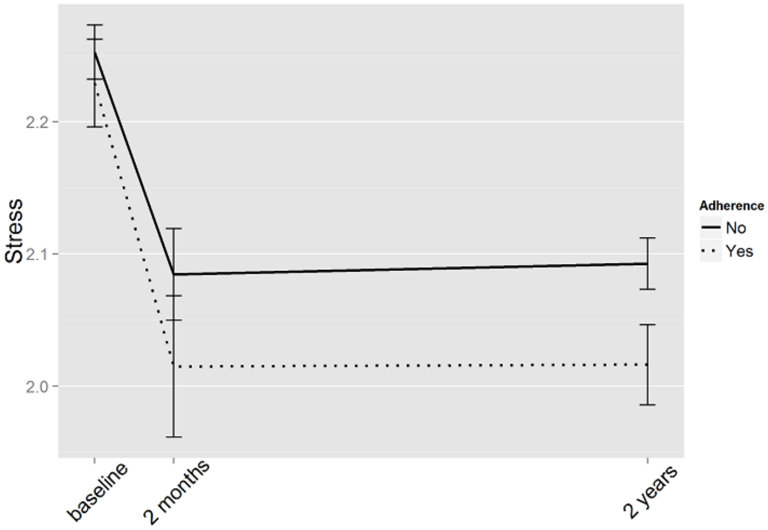

Supplementary Figure 6. Confidence in the future in the intervention group by adherence to the treatment protocol

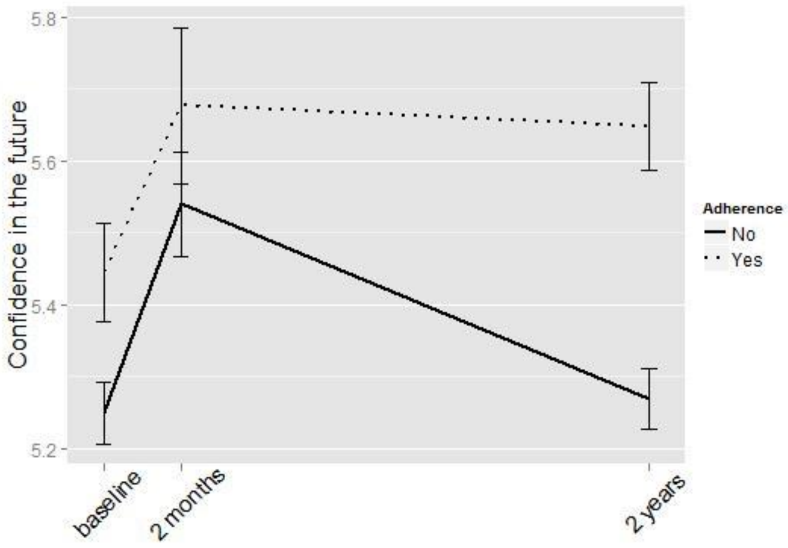

Supplementary Figure 7. Gratitude in the intervention group by adherence to the treatment protocol

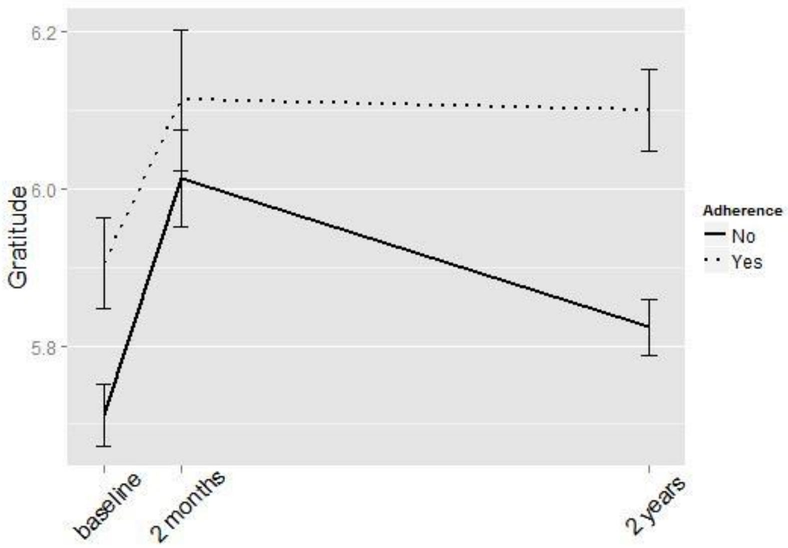

Supplement: Additional file 1: Table S1. — The themes of the emails. Table S2. Participation for different types of interventions. Table S3. Outcome variables in the intervention and controls groups at baseline, 2-month and 2-year follow-ups. Figure S1. Binge drinking in the intervention and controls groups over time. Figure S2. Daily smoking in the intervention and control groups over time. Figure S3. The proportion of participants doing physical exercise at least 3 h per week in the intervention and control groups over time. Figure S4. The proportion of participants using vegetables or fruits daily in the intervention and control groups over time. Figure S5. Level of stress in the intervention group by adherence to the treatment protocol. Figure S6. Confidence in the future in the intervention group by adherence to the treatment protocol. Figure S7. Gratitude in the intervention group by adherence to the treatment protocol. (PDF 302 kb) [file 40359_2016_125_MOESM1_ESM.pdf]
